# Prostate cancer in *BRCA2* germline mutation carriers is associated with poorer prognosis

**DOI:** 10.1038/sj.bjc.6605822

**Published:** 2010-08-24

**Authors:** S M Edwards, D G R Evans, Q Hope, A R Norman, Y Barbachano, S Bullock, Z Kote-Jarai, J Meitz, A Falconer, P Osin, C Fisher, M Guy, S G Jhavar, A L Hall, L T O'Brien, B N Gehr-Swain, R A Wilkinson, M S Forrest, D P Dearnaley, A T Ardern-Jones, E C Page, D F Easton, R A Eeles, The UK Genetic Prostate Cancer Study Collaborators and BAUS Section of Oncology

**Affiliations:** 1 Oncogenetics team, Section of Cancer Genetics, Institute of Cancer Research, Sutton SM2 5PT, UK; 2 St Mary's Hospital, CMFT, Oxford Road, Manchester, M13 9WL, UK; 3 The Royal Marsden NHS Foundation Trust, London & Sutton, SM2 5PT, UK; 4 Team 67, The Wellcome Trust Sanger Institute, Cambridge, CB10 1SA, UK; 5 Cancer Research UK Genetic Epidemiology Unit, Strangeways Research Labs, Cambridge, CB1 8RN, UK

**Keywords:** prostate cancer, *BRCA2*, prognosis, genetic testing

## Abstract

**Background:**

The germline *BRCA2* mutation is associated with increased prostate cancer (PrCa) risk. We have assessed survival in young PrCa cases with a germline mutation in *BRCA2* and investigated loss of heterozygosity at *BRCA2* in their tumours.

**Methods:**

Two cohorts were compared: one was a group with young-onset PrCa, tested for germline *BRCA2* mutations (6 of 263 cases had a germline *BRAC2* mutation), and the second was a validation set consisting of a clinical set from Manchester of known *BRCA2* mutuation carriers (15 cases) with PrCa. Survival data were compared with a control series of patients in a single clinic as determined by Kaplan–Meier estimates. Loss of heterozygosity was tested for in the DNA of tumour tissue of the young-onset group by typing four microsatellite markers that flanked the *BRCA2* gene, followed by sequencing.

**Results:**

Median survival of all PrCa cases with a germline *BRCA2* mutation was shorter at 4.8 years than was survival in controls at 8.5 years (*P*=0.002). Loss of heterozygosity was found in the majority of tumours of BRCA2 mutation carriers. Multivariate analysis confirmed that the poorer survival of PrCa in *BRCA2* mutation carriers is associated with the germline *BRCA2* mutation *per se.*

**Conclusion:**

*BRCA2* germline mutation is an independent prognostic factor for survival in PrCa. Such patients should not be managed with active surveillance as they have more aggressive disease.

Prostate cancer (PrCa) is a significant public health problem. In the European Union, approximately 200 000 men are diagnosed annually with the disease. There are 35 515 cases (Cancer Research UK, 2009a) per year in the United Kingdom and 10 239 deaths (Cancer Research UK, 2009b). It is now the commonest male non-cutaneous cancer diagnosed in the United Kingdom; the lifetime risk of being diagnosed with PrCa is 1 in 10 (Cancer Research UK, 2009a). Although the increase in population screening is leading to an increase in diagnosis, many men will not develop aggressive disease. However, it is recognised that some PrCa cases have a particularly poor prognosis. Although there are some histological and stage predictors of prognosis (Kattan and Scardino, 2002), until recently, none of them have been related to inherited factors.

Multiple aetiologies have been proposed to contribute to the development of PrCa. There is strong evidence that inherited genetic factors are important and exhibit significant familial aggregation in some men, particularly when affected at a young age (Woolf, 1960; Edwards and Eeles, 2004). There is a recognised association of breast cancer with PrCa in families (Thiessen, 1974; Anderson and Badzioch, 1992; Tulinius *et al*, 1992). Male relatives in breast cancer families in Iceland have a 2–3-fold risk of PrCa (Sigurdsson *et al*, 1997). The breast cancer predisposition genes *BRCA1* and *BRCA2* have been reported to increase the risk of PrCa by three-fold and seven-fold, respectively, in male mutation carriers ascertained through a family history of breast cancer (Ford *et al*, 1994; Struewing *et al*, 1997; Breast Cancer Linkage Consortium, 1999). Analyses of PrCa relative risks (RR) in male mutation carriers in breast cancer families from the Breast Cancer Linkage Consortium showed an RR of 4.65 (95% CI: 3.48–6.22) of PrCa in male *BRCA2* mutation carriers (the RR is 7.33 below the age of 65 years) and of 1.07 (0.75–1.54) in *BRCA1* carriers (with an RR of 1.82 (1.01–3.29) for men under 65 years of age) (Thompson and Easton, 2001, 2002). The estimated cumulative incidence of PrCa by the age of 70 years is 7.5–33%. Recent studies have suggested that the risk of PrCa in *BRCA2* mutation carriers may be as high as an RR of 23-fold at age 60 years (Edwards *et al*, 2003). Studies from Iceland have reported that germline mutations in *BRCA2* may be involved not only in susceptibility to PrCa but also in the aggressiveness of the disease (Sigurdsson *et al*, 1997; Tryggvadottir *et al*, 2007). However, these individuals all carried a common founder mutation (999del5 in *BRCA2*). Narod *et al* (2008) have reported that PrCa survival in *BRCA2* mutation carriers is much shorter (median survival from diagnosis was 4 years) when compared with *BRCA1* carriers’ survival (median survival from diagnosis was 8 years). In PrCa, in which the *BRCA2* germline mutation status was unknown, allele loss at the *BRCA2* locus has been shown to be a prognostic factor for survival on univariate analysis (Edwards *et al*, 1998), and loss of the wild-type allele would imply a tumour suppressor mechanism in predisposition to this disease in *BRCA2* mutation carriers, but it is not known whether this is a surrogate for high grade or is due to mutation *per se* (Knudson, 1971; Willems *et al*, 2008).

A *BRCA2* genomic screening study has previously been undertaken by ourselves, and six potentially pathogenic germline *BRCA2* mutations were found in a set of 263 PrCa patients diagnosed at ⩽55 years (2.3%) (Edwards *et al*, 2003). In this study we report clinical follow-up data and the results of loss of heterozygosity (LOH) analyses on PrCa tumours from the mutation carriers in this report.

We then studied a second validation data set of men with germline mutations in the *BRCA2* gene from a cancer genetics clinic and assessed their survival to confirm our results in a different UK data set of male *BRCA2* mutation carriers with PrCa.

## Materials and methods

### Patient recruitment and survival analyses

Two groups of men with PrCa were studied.

1) *A series of men with PrCa from the UK Genetic Prostate Cancer Study (UKGPCS):*

Patient recruitment was conducted as reported in a previous article (Eeles *et al*, 1997). The coding region of *BRCA2* was analysed from blood DNA from 263 PrCa patients diagnosed at ⩽55 years and germline mutations in *BRCA2* were found in 6 men (2.3%) (Edwards *et al*, 2003). The control group consisted of men from a systematic series of prostate cancer patients, age and stage matched from our prostate cancer clinic (1587 cases). The majority of patients had clinically presenting (non-screen detected) disease at diagnosis. Clinical data were collected on survival/date of last follow-up for both deleterious *BRCA2* mutation carriers and controls. Supplementary Table 1 shows the demographic and clinical characteristics of prostate cancer in this group of patients, for both the cases and controls.

2) *Men with PrCa who also harbour a germline BRCA2 mutation from a clinical series:*

Men attending a cancer genetics clinic in Manchester, who were found on clinical genetic testing to harbour *BRCA2* mutations, were reviewed from the Access clinical database and their date of death or last follow-up was ascertained from the cancer registry or from their clinical notes.

Written informed consent was obtained from individuals in this study (ethics number 06/MRE02/4).

Overall survival was measured from date of diagnosis to date of death or last follow-up. Kaplan–Meier survival analyses were undertaken with patients censored at date of last follow-up. The overall survival of those with and without germline mutations in *BRCA2* in group 1 was compared using the log-rank test. The overall survival for those in group 2 was also calculated separately and in combination with group 1. The effect of other factors that could affect survival was analysed using Cox regression. The factors investigated were stage at diagnosis, incidental PSA detection, Gleason score, grade, whether they had a prostatectomy, PSA at diagnosis and age.

### DNA extraction and LOH studies

Germline DNA was obtained from peripheral blood samples and extracted as reported in previous articles (Edwards *et al*, 1997). Tumour DNA was obtained from microdissected formalin-fixed paraffin-embedded (FFPE) blocks and extracted as reported in previous articles (Edwards *et al*, 1998). Normal tissue DNA was also obtained in the same way.

Four microsatellite markers, D13S260, D13S171, D13S267 and D13S1493 within and flanking the *BRCA2* gene, were typed on five tumours from those men in group 1 using an ABI (Applied Biosystems, Life Technologies Corporation, Carlsbad, CA, USA) 377 Genetic Analyser. The ‘peak height’ of the alleles was used to determine the ratio of allelic loss compared with either genomic DNA or adjacent normal tissue from paraffin blocks. Percentage allele loss for informative markers (minimum of two) was averaged for each patient.

To determine which allele was lost in the microsatellite LOH results, a sequencing method was used as described in Boettger *et al* (2003). We used Applied Biosystems dRhodamine chemistry on a 377 Genetic Analyser (Edwards *et al*, 2003). An average value for the loss was estimated by examining a number of electropherogram peak height signals in the area of the mutation.

## Results

### Survival analysis

**Figure 1:**
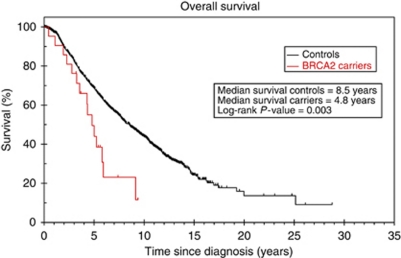
Kaplan–Meier survival estimates of 21 *BRCA2* mutation carriers.

The median overall survival of all *BRCA2* mutation carriers was significantly shorter at 4.8 years compared with that of non-carriers at 8.5 years; log rank *P*=0.003 (hazard ratio 2.14 (95% CI: 1.28–3.56); see Figure 1). When analysed by method of ascertainment (see methods), the median survival of the six *BRCA2* carriers in group 1 was significantly shorter at 3.6 years (*P*=0.002; hazard ratio 3.36 (95% CI: 1.50–7.50)), when compared with that of non-carriers. In group 2, the 15 men with germline *BRCA2* mutations and PrCa had a median survival of only 5.0 years.

The mutations in *BRCA2* in the men with PrCa are listed in Table 1.

Table 2 shows the univariate results. This shows that the following factors are associated with a poorer overall survival: germline *BRCA2* mutation status, tumour (T), nodal (N) and metastasis (M) stage, tumour detected clinically rather than by PSA screening, higher Gleason score, treatment that did not involve prostatectomy, PSA at diagnosis of ⩾25 ng ml^−1^ and age >55 years at diagnosis. In a multivariate analysis, which is shown in Table 3, germline *BRCA2* mutation status, T and nodal (N) tumour stage, higher grade, treatment that did not involve prostatectomy, PSA at diagnosis of ⩾25 ng ml^−1^ and higher age at diagnosis remained independent prognostic factors.

### Loss of heterozygosity (LOH) results

**Figure 2:**
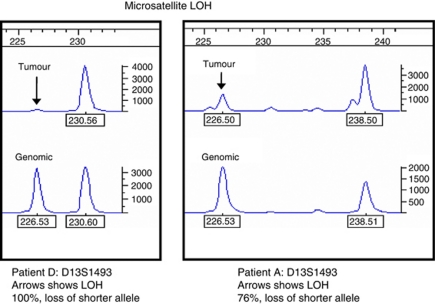
Example of LOH seen with D13S1493 in two patients – A and D.

The DNA from microdissected FFPE tumour tissue was available from 5 of 6 germline *BRCA2* carriers. All five showed LOH (see Table 4). A representative microsatellite trace is shown in Figure 2.

**Figure 3:**
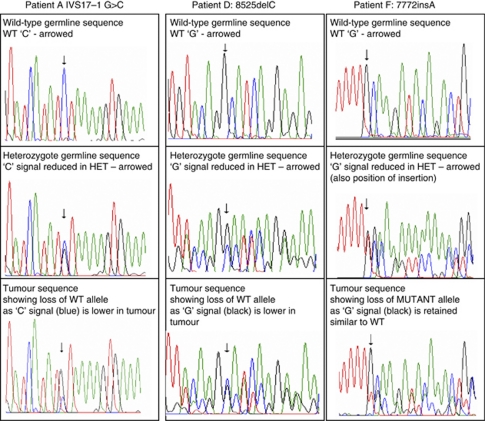
Examples of allele loss in patients A, D and F. Arrows show the position of a representative peak for signal analysis.

To estimate which allele was lost, a sequencing method was adopted. Of the five tumour samples sequenced, it was observed that patient F had lost the mutant allele, whereas patients A, D and E lost the wild-type allele. The tumour from patient B had lost the mutant allele at a low level (approx 10% Table 4). Representative sequence traces are shown in Figure 3.

## Discussion

We have shown that men with PrCa, who also harbour a deleterious germline mutation in the *BRCA2* gene, have a poorer overall survival. This has been shown in a small sample of men who were diagnosed at ⩽55 years with PrCa, compared with those diagnosed at a similarly young age, but who did not harbour a germline mutation as determined by coding sequence analysis from a previous study, and also men from a systematic series of prostate cancer cases in one centre: group 1 (Edwards *et al*, 2003). We have validated this in a separate data set of men who have had PrCa at any age and who have been found to have a germline mutation in *BRCA2* by a clinical genetic testing service, in which genetic testing was offered as part of genetic counselling of families with mutations: group 2. These are usually men within breast cancer families in which women have initially been tested to determine the cause of familial breast cancer clustering within the family. Again, they have a poorer prognosis than men who do not harbour a germline *BRCA2* mutation.

It has been shown that local extent or T stage, N stage and presence or not of M stage and higher PSA at presentation are all predictors of poorer survival (Kattan and Scardino, 2002). It was therefore very important to determine whether the poorer survival associated with the presence of a germline *BRCA2* mutation was independent. The multivariate analysis confirms that the presence of a germline *BRCA2* mutation is a marker of poorer overall survival *per se.*

This has implications for the detection and management of men with PrCa who are found to harbour germline mutations in the *BRCA2* gene, as their poorer survival would be a contraindication for active surveillance. It is not yet known whether these men should have a particular modality of treatment (e.g., surgery rather than radiation), as the sample sizes in this paper are too small and treatment data are incomplete in these data sets to be able to determine this.

Before 2008, the only data available on survival in men with PrCa who harbour germline mutations in *BRCA2* were from Iceland, where there is a founder mutation (Thorlacius *et al*, 1996; Sigurdsson *et al*, 1997; Tryggvadottir *et al*, 2007). Tryggvadottir *et al* (2007) found that PrCa carriers with the *BRCA2* 999del5 mutation had a lower mean age at diagnosis, more advanced PrCa as assessed by stage and grade, and a shorter median survival time compared with non-carriers. Their study showed a median survival time for carriers (30) of 2.1 years, which was significantly shorter than the 12.4 years for non-carriers (497). The survival time of the Icelandic carriers is much shorter than that reported for our early-onset group 1 carriers of 4 years. One factor to be noted in the Tryggvadottir *et al* (2007) study is that all of the 527 PrCa patients were related to breast cancer patients, 28% of whom were first-degree relatives. Of the 527 patients, 30 were determined to be 999del5 carriers. It is therefore possible that the poorer prognosis that was reported could have pertained only to this specific mutation type. This result, and its potential specificity by virtue of mutation and population, has been discussed further by Boormans and Schröder (2007). In contrast, we have shown that in the UK population, men with a variety of other mutations in *BRCA2*, are likely to have a similarly poorer survival, and therefore the poorer survival is likely to be related to different deleterious mutations in the *BRCA2* gene.

We found LOH in the five available PrCa tumours from men in group 1.Three of the five tumours had lost the wild-type allele. This is consistent with a tumour suppressor model and indicative of a causal relationship between *BRCA2* germline mutations and predisposition to PrCa in these individuals. Loss of the mutant allele was also observed. The implication is that for disease causation, maybe a gene dosage effect is important.

Tommiska *et al* (2008) have recently proposed a model of ‘conditional haploinsufficiency’, whereby defects in genes such as *BRCA1* or *BRCA2* can disrupt the regulation of other important genome integrity monitoring genes such as *ATM*. In this hypothesis, the local effects of these predisposing genes, by virtue of perhaps increased DNA double-strand breaks, would ultimately cause ATM protein inactivation as the cells progressed to malignancy. Although this haploinsufficiency mechanism has only been investigated in breast cancer, it may have implications for the development of PrCa in the carriers that we studied, and could explain why we saw loss of mutant alleles in comparison with the classical loss of wild type.

There is very scant literature on the ‘classical’ loss of wild-type alleles from PrCa patients who are *BRCA2* carriers. Gudmundsson *et al* (1995) and Grönberg *et al* (2001) have reported LOH in PrCa patients. Gudmundsson *et al* (1995) investigated five high-risk breast cancer families and found seven men with PrCa, six of whom had LOH at the *BRCA2* locus. Grönberg *et al* (2001) studied a breast/prostate family (three breast cancer, five PrCa) that was found to have a deleterious *BRCA2* mutation (6051delA). Of the four brothers with PrCa, two had LOH (loss of WT) and two retained heterozygosity.

Willems *et al* (2008) reported on the screening of a large series of kConFab Australian *BRCA2* breast cancer families. There were many men with PrCa in these families; however, 20 were confirmed to be *BRCA2* carriers, and 14 of them had tumours that were available for analysis by multiplex ligation-dependent probe amplification (MLPA). This technique is able to assay the entire *BRCA2* gene for loss of promoter and coding regions. Of the 14 *BRCA2* carriers, 10 showed loss of heterozygosity by the MLPA method. The set comprised six men with a known family history of PrCa and all the six showed LOH at *BRCA2*. The conclusion was that the wild-type allele was most often lost, but for the four cases that showed no LOH, epigenetic and haploinsufficiency models were postulated as possible mechanisms. This allele determination was assessed by a sequencing technique that is similar to the one we used. Willems *et al* (2008) reported comprehensive clinical data on their carriers and it is noteworthy that all 10 PrCa cases presented with high Gleason scores of 9. This is a finding that is similar to the high-grade presentation of the men in our study group 1. The risk estimates of 3.5-fold for PrCa, as reported by Willems *et al* (2008), are lower than the RR reported by us in 2003 (Edwards *et al*, 2003).

In 2008, Narod *et al* (2008) reported data on a panel of PrCa patients with *BRCA1* and *BRCA2* germline mutations identified from breast cancer families. For the combined group of known and inferred carriers, the median survival for 183 *BRCA2* patients was 4.0 years *vs* 8.0 years for the 119 men in the *BRCA1* group. When only known carriers were analysed, the results were 5.0 years (67 *BRCA2* known carriers) and 15.0 years (37 *BRCA1* known carriers). Although there is a difference in the survival of *BRCA1* patients, which is discussed by the authors, the poorer survival of *BRCA2*-related patients is not in dispute. Use of either survival measure, 4 or 5 years, illustrates that the median survival of the patients studied is very similar to that observed in our study of 4.8 years.

It is not yet known whether earlier detection of PrCa in men with germline mutations in *BRCA2* will result in a better outcome and whether PSA screening is suitable for such a population. The IMPACT study (Identification of Men with a genetic predisposition to ProstAte Cancer: Targeted screening in *BRCA1* and *BRCA2* mutation carriers and controls) has been developed to investigate the role of targeted PrCa screening in male *BRCA1* and *BRCA2* gene mutation carriers using an annual PSA screen (Mitra *et al*, 2007). Early data have suggested that men will uptake screening and that PrCa was twice as likely in *BRCA1/2* mutation carriers (Horsburgh *et al*, 2005).

Our data have shown that the observation in Iceland, which shows that men who carry the Icelandic founder mutation in the *BRCA2* gene who also develop PrCa have a poorer survival, is not restricted to this mutation and we have shown that this is due to the presence of a deleterious mutation within the *BRCA2* gene *per se.* There is some dispute about the precise frequency of germline *BRCA2* mutation in men with PrCa. This is reported to be about 1% in men aged ⩽55 years in a US series (Agalliu *et al*, 2007), but this may be equivalent to 65–68 years at *clinical* diagnosis, as the prevalence of PSA screen-detected disease is higher in the United States. This is likely to be the case, as in this US series, 67.3% of men had a PSA of <10. We have found that 2.3% of men diagnosed with clinically presenting disease (non-PSA detected) have a germline *BRCA2* mutation (Edwards *et al*, 2003). Even if the incidence of a germline mutation in men with young-onset (defined as ⩽55 years at diagnosis) PrCa was as low as 2%, the finding of a *BRCA2* mutation would be an indication to avoid active surveillance in these patients, as they have a more aggressive disease outcome.

## Figures and Tables

**Table 1:** List of germline mutations in *BRCA2* men with prostate cancer from group 1 (diagnosed at age 55 years or under) and group 2

**Patient ID**	**Age at diagnosis (Years)**	**BRCA2 mutation (nt) U43746**	**BRCA2 mutation (nt) NM_000059.1**	**Codon U43746**	**Codon NM_000059.1 with HGVS nomenclature**
Group 1 patient A	44	8205-1g>c (IVS17-1g>c)	7977-1g>c	IVS17-1 g>c (splice site)	7977-1g>c (splice site)
Group 1 patient B	47	7084delAAAAG	6856delAAAAG	Stop 2291	Arg2287LeufsX4
Group 1 patient C	48	2558insA or 2558dupA	2330insA or 2330dupA	Stop 787	Asp777GlufsX11
Group 1 patient D	52	8525delC	8297delC	Stop 2776	Thr2766AsnfsX11
Group 1 patient E	52	6714delACAA^*^	6486delACAA	Stop 2166	Lys2162AsnfsX5
Group 1 patient F	53	7771insA or 7771dupA	7543insA or 7543dupA	Stop 2537	Thr2515AsnfsX24
					
Group 2 patient 1	66	6174delT	5946delT	Stop 2003	Ser1982ArgfsX22
Group 2 Patient 2	66	5910C>G	5682C>G	Y1894X (Tyr to Stop)	Tyr1894X
Group 2 patient 3	79	9610C>T	9382C>T	R3128X (Arg to Stop)	Arg3128X
Group 2 patient 4	74	860-1g>a	632-1g>a	IVS7-1g>a (splice site)	632-1g>a (splice site)
Group 2 patient 5	46	6503delTT (+) 10204A>T	6275-6276delTT (+) 9976A>T	Stop 2098 + K3326X	Leu2092ProfsX7 + Lys3326X
Group 2 patient 6	77	2157delG	1929delG	Stop 659	Arg645GluX15
Group 2 patient 7	74	6503delTT (+) 10204A>T	6275-6276delTT (+) 9976A>T	Stop 2098 + K3326X	Leu2092ProfsX7 + Lys3326X
Group 2 patient 8	57	6819delTG	6591delTG	Stop 2201	Glu2198AsnfsX4
Group 2 patient 9	59	3036delACAA	2808delACAA	Stop 959	Ala938ProfsX21
Group 2 patient 10	72	6137C>A	5909C>A	S1970X	Ser1970X
Group 2 patient 11	62	2117delC	1889delC	Stop 643	Thr630AsnfsX14
Group 2 patient 12	74	del exons 14-16	94189-?_97257+?del	Del exons 14-16	del exons14_16
Group 2 patient 13	66	del exons 14-16	94189-?_97257+?del	Del exons 14-16	del exons14_16
Group 2 patient 14	56	5950delCT	5722delCT	Stop 1909	Leu1908ArgfsX2
Group 2 patient 15	73	2157delG	1929delG	Stop 659	Arg645GluX15

**Table 2:** Univariate analysis of overall survival

**Factor**	**Group**	**Number of patients**	**Hazard ratio (95% CI)**	***P*-value**
*BRCA2* mutation carrier	No	1587	1.00	0.003
	Yes	21	2.14 (1.28–3.56)	
Clinical T stage	T1	165	1.00	<0.001
	T2	409	1.23 (0.92–1.65)	
	T3	510	1.88 (1.43–2.49)	
	T4	152	3.87 (2.83–5.28)	
	TX	325	4.80 (3.63–6.36)	
N stage	N0	882	1.00	<0.001
	N1–3	165	3.04 (2.46–3.76)	
	NX	514	3.32 (2.88–3.84)	
M stage	M0	1008	1.00	<0.001
	M1	456	4.38 (3.80–5.05)	
	MX	86	1.61 (1.21–2.15)	
Incidental PSA detection	Yes	127	1.00	<0.001
	No	1362	2.41 (1.74–3.34)	
Gleason score	⩽7	686	1.00	<0.001
	>7	181	2.30 (1.85–2.85)	
Grade	1	200	1.00	<0.001
	2	857	1.77 (1.38–2.27)	
	3	330	4.02 (3.09–5.23)	
Prostatectomy	Yes	82	1.00	<0.001
	No	1423	4.70 (2.82–7.83)	
PSA at diagnosis	<25	498	1.00	<0.001
	⩾25	487	2.19 (1.83–2.63)	
Age group	⩽55	119	1.00	0.02
	>55	1474	1.40 (1.05–1.87)	
Age	Per year	1593	1.05 (1.04–1.06)	<0.001

Abbreviations: CI=confidence interval; M=metastasis; N=nodal; PSA=prostate-specific antigen; T=tumour.

**Table 3:** Multivariate analysis of overall survival (significant factors only)

**Factor**	**Group**	**Number of patients**	**Number of events**	**Hazard ratio (95%CI)**	***P*-value**
*BRCA2* mutation carrier	No	506	218	1	0.002
	Yes	3	3	7.54 (2.11–26.98)	
Clinical T stage	T1	51	16	1	0.002
	T2	158	49	0.99 (0.56–1.75)	
	T3	202	87	1.19 (0.68–2.05)	
	T4	50	34	1.87 (1.00–3.48)	
	TX	48	35	2.34 (1.24–4.40)	
N stage	N0	344	111	1	<0.001
	N1–3	66	40	2.01 (1.36–2.98)	
	NX	99	70	3.72 (2.65–5.23)	
Grade	1	33	7	1	<0.001
	2	376	152	2.24 (1.03–4.88)	
	3	100	62	3.94 (1.78–8.73)	
Prostatectomy	Yes	31	4	1	0.044
	No	478	217	2.84 (1.03–7.85)	
PSA at Diagnosis	<25	282	96	1	0.027
	⩾25	227	125	1.39 (1.04–1.86)	
Age	Per year>	509	221	1.02 (1.00–1.04)	0.026

Abbreviations: CI=confidence interval; N=nodal; PSA=prostate-specific antigen; T=tumour.

**Table 4:** LOH frequencies observed for the five patients from group 1

**Patient ID**	**Number of informative markers**	**LOH (%)** a	**Allele lost** b
A	3	77	WT
B	4	87	MUT
D	2	97	WT
E	4	77	WT
F	3	81	MUT

Abbreviations: MUT=mutant; LOH=loss of heterozygosity; WT=wildtype.

a LOH (%) by microsatellite analysis (average).

b Allele loss estimation by sequencing.
